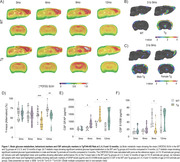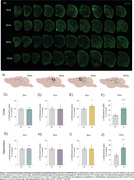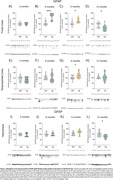# Astrocyte reactivity and cerebral glucose hypermetabolism in early Alzheimer's: insights from a longitudinal study in the TgF344‐AD rat model

**DOI:** 10.1002/alz.094050

**Published:** 2025-01-09

**Authors:** Amanda Muliterno Domingues Lourenço de Lima, Andreia Silva da Rocha, Luiza Santos Machado, Carolina Soares, Pamela C.L. Ferreira, Bruna Bellaver, Patrícia Eduarda Carvalho da Silva, Gianina Teribele Venturin, Samuel Greggio, Gabriela Lazzarotto, Pedro Vidor, Peter Kunach, Kim N. Green, Jaderson Costa da Costa, Diogo O. Souza, Pedro Rosa‐Neto, Eduardo R. Zimmer, MODEL AD

**Affiliations:** ^1^ Universidade Federal do Rio Grande do Sul, Porto Alegre, Rio Grande do Sul Brazil; ^2^ University of Pittsburgh, Pittsburgh, PA USA; ^3^ Universidade Federal de Ciências da Saúde de Porto Alegre, Porto Alegre, RS Brazil; ^4^ Brain Institute of Rio Grande do Sul, Porto Alegre Brazil; ^5^ McGill University, Montreal, QC Canada; ^6^ University of California, Irvine, Irvine, CA USA; ^7^ Brain Institute of Rio Grande do Sul ‐ Pontifícia Universidade Católica do Rio Grande do Sul, Porto Alegre, Rio Grande do Sul Brazil; ^8^ Universidade Federal do Rio Grande do Sul, Porto Alegre Brazil

## Abstract

**Background:**

Recent studies have suggested a transient glucose hypermetabolism in early phases of Alzheimer's Disease (AD), which is followed by a characteristic glucose hypometabolism in dementia stages. This phenomenon desveres further investigation and it is suggested to be associated to glial/inflammatory or compensatory neuronal responses. Here, we aimed to longitudinally investigate brain glucose metabolism in an AD animal model and explore associated cellular and inflammatory changes.

**Method:**

Longitudinal assessments, including cerebral glucose metabolism (18F]FDG‐PET), behavioral tasks and cerebrospinal fluid (CSF) sample collection were performed at 3, 6, 9, and 12 months of age (mo) in wild type (WT) and TgF344‐AD rats (Tg). Glial and inflammatory markers were evaluated in the CSF via ELISA multiplex. A cross‐sectional cohort was used to follow the spatial distribution of Aß plaques (IHC), to assess the brain content of glial and neuronal proteins (western blot), and to analyze the cortical glutamate uptake (ex‐vivo slices) at the same time points.

**Result:**

At 9mo, three months after the initial appearance of Aß plaque deposits, Tg animals exhibited cortical glucose hypermetabolism (Figure 1A‐C). At the same age, astrocytic glutamate uptake was increased in the cortex and hippocampus (Figure 2). Declines in performance on the Y‐maze and Novel Object Recognition tasks were observed at 9 and 12mo (Figure 1D). CSF analysis revealed elevated GFAP levels at 6, 9 and 12mo, and reduced S100B at 9mo (Figure 1E e F). Tissue GFAP immunocontent increased in the temporoparietal cortex at 6 and 9mo, in the hippocampus at 9mo, and was reduced in the frontal and temporoparietal cortices at 12mo (Figure 3).

**Conclusion:**

Our findings suggest the presence of an early, transient phase of brain glucose hypermetabolism in TgF344‐AD rats, consistent with observations in recent animal and human studies. This phenomenon seems to be closely linked to the astrocyte response, reflected in variations of crucial astrocyte proteins such as GFAP and S100B, along with an increase in the glutamate uptake by these cells. In contrast, neuronal, microglial and inflammatory markers did not exhibit changes during this timeframe.